# Emulsion Cross-Linking Technique for Human Fibroblast Encapsulation

**DOI:** 10.1155/2018/9317878

**Published:** 2018-07-11

**Authors:** Watcharaphong Chaemsawang, Weerapong Prasongchean, Konstantinos I. Papadopoulos, Suchada Sukrong, W. John Kao, Phanphen Wattanaarsakit

**Affiliations:** ^1^Department of Pharmaceutics and Industrial Pharmacy, Faculty of Pharmaceutical Sciences, Chulalongkorn University, Bangkok, Thailand; ^2^Department of Biochemistry and Microbiology, Faculty of Pharmaceutical Sciences, Chulalongkorn University, Bangkok, Thailand; ^3^THAI StemLife Co., Ltd., Bangkok, Thailand; ^4^Department of Pharmacognosy and Pharmaceutical Botany, Faculty of Pharmaceutical Sciences, Chulalongkorn University, Bangkok, Thailand; ^5^Chemistry and Biology Centre, Li Ka Shing Faculty of Medicine and Faculty of Engineering, The University of Hong Kong, Hong Kong SAR, Hong Kong

## Abstract

Microencapsulation with biodegradable polymers has potential application in drug and cell delivery systems and is currently used in probiotic delivery. In the present study, microcapsules of human fibroblast cells (CRL2522) were prepared by emulsion cross-linking technique. Tween 80 surfactant at a 2% concentration through phase inversion resulted in the most efficient and stable size, morphology, and the cells survival at least 50% on day 14. Emulsion cross-linking microcapsule preparation resulted in smaller and possibly more diverse particles that can be developed clinically to deliver encapsulated mammalian cells for future disease treatments.

## 1. Introduction

Microencapsulation using biodegradable polymers has potential application in drug and cell delivery systems [1–4]. Coacervation, solvent evaporation, and spray drying are examples of technique used in producing microcapsules that are robust enough to withstand external forces and allowing them to be implanted using needles or catheters for drug delivery to target organs. Another commonly used encapsulation method is ionic gelation that does not require heat or organic solvents but the size of particles is larger and difficult to use in the clinic [5–7]. High shear speed cutter and ultrasonic and spray guns can be used to reduce particle size of ionic gelation microcapsules. Microencapsulation by emulsion cross-linking is an easier technique to achieve microcapsule size reduction. Emulsion cross-linking is a favored method in probiotic and other biological product encapsulation [5]. The objective of this study is to develop emulsion cross-linking encapsulation for cell delivery. How surfactant type and concentration and oil aqueous phase ratio impacted particle size, stability, and number as well as cell viability in the resulting encapsulation was examined.

## 2. Materials and Methods

### 2.1. Chemicals and Reagents

Sodium alginate was purchased from Sigma–Aldrich (CAS number 9005-38-3). Calcium chloride was purchased from Merck. Lecithin (phosphatidyl choline S75) was obtained as a gift sample from Lipoid. Tween 80 and Span 80 were purchased from Srichand United Dispensary.

### 2.2. Cell Culture

Human fibroblast cells (CRL-2522ATCC) were cultured in high glucose Dulbecco's modified Eagle's medium (DMEM: containing 10 % fetal bovine serum and 1% penicillin-streptomycin- amphotericin B). Cells were cultured at 7°C with 5% CO_2_ and the medium was replenished every three days. Cells were dissociated with trypsin–EDTA and were enumerated with trypan blue under microscope. The cells passage of 20-30 was used for microencapsulation. All of medium materials were purchased from Invitrogen.

### 2.3. Development of Alginate Microcapsules by Emulsion Cross-Linking Technique

Water-in-oil (W/O) emulsion was prepared by mixing 1% sodium alginate solution with rice bran oil. A 1% sodium alginate solution was generated by dissolving 1 g of alginate (Sigma–Aldrich) in 100 ml cell culture medium. Rice bran oil and surfactant were mixed with a magnetic stirrer. The alginate solution was mixed in the rice bran oil solution and stirred for 10 minutes. In the second stage the primary emulsion was rinsed in 2% calcium chloride solution and continuously stirred for 20 minutes. Then, the microcapsules were centrifuged at 2,000 rpm for 20 minutes and washed three times with PBS pH 7.4. The effect of rice bran oil, aqueous phase ratio, surfactant type, and concentration of surfactant on the resulting microcapsules, was examined.

### 2.4. Cell Encapsulation in Alginate Microcapsule by Emulsion Cross-Linking Technique

Human fibroblast cells at a concentration of 5x10^5^ cells/ml were placed in 1 % (w/v) sodium alginate solution and transferred to the oil solution and mixed for 10 minutes. The cell suspension was placed into calcium chloride bath and stirred for 20 minutes at room temperature. The resulting fibroblast-containing microcapsules were kept in DMEM medium and incubated at 37°C with 5% CO_2_. The cell culture medium was washed and changed every 3^rd^ day.

### 2.5. Characterization of Microcapsules

The size and morphology of the microcapsules were determined under inverted microscope and Malvern mastersizer. Percent living cell entrapment was calculated from

(1) Living cell entrapment=Number of living cell in microcapsuleNumber of living cell loadingx100

### 2.6. Assessment of Encapsulated Cells Viability

Number of living cell in microcapsule was determined by fluorimetric quantitative PrestoBlue^®^ assay. PrestoBlue reagent, a solution of resazurin base, is rapidly taken up by living cells. The reducing environment within viable cells converts PrestoBlue reagent to an intensely red-fluorescent dye which was analyzed using microplate reader (Perkin Elmer) at excitation 560, emission 590 nm.

## 3. Results and Discussion

### 3.1. Development of Microencapsulation

**Figure 1:**
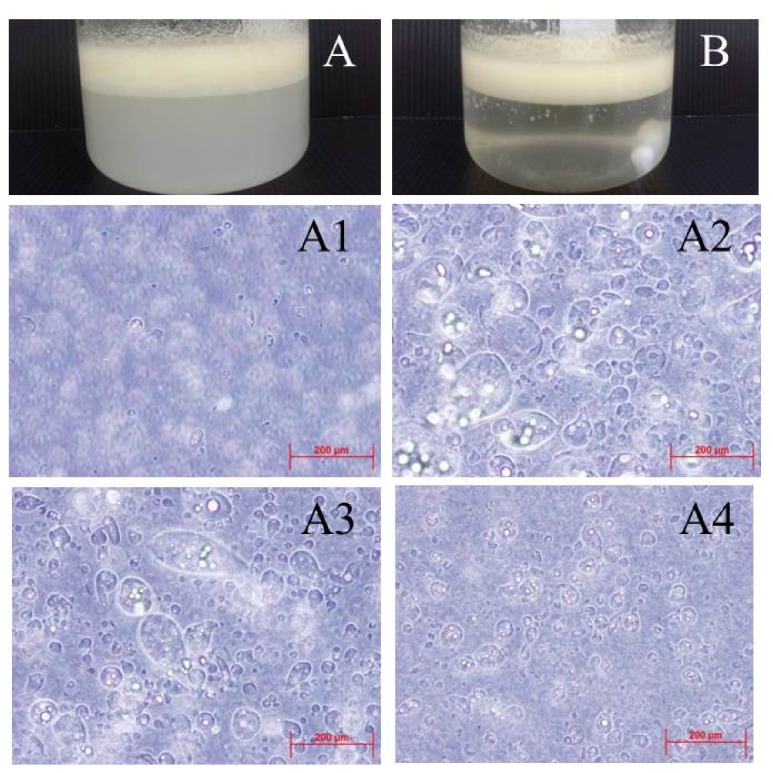
Microencapsulation. Prepared microcapsules with (A) Tween 80 and (B) without surfactant. The emulsion or oil layer is seen in the upper white opaque area while the calcium chloride solution is the clearer area under the oil layer. The picture shows the effect on microcapsule morphology under microscope of (A1) 1% Tween 80, (A2) 2% Tween 80 (A3) 3% Tween 80, and (A4) 5% Tween 80.

The microcapsules were prepared by emulsion crosslink using variable types and concentrations of surfactants. We used Tween 80, Span 80, and Lecithin to investigate the effects of surfactants and while no surfactant preparation was used as the control. Microencapsulation using 2% Tween 80 (Figure 1(A)) led to a turbid calcium chloride solution from suspended microcapsules in the solution. In the absence of surfactant (Figure 1(B)) no microcapsules could be produced as the emulsion was not stable and thus the calcium chloride solution remained clear. The aqueous phase separated directly from emulsion when the stir is stopped due to the lack of surfactant and the non-homogenous hydrophilic and hydrophobic portions.

**Figure 2:**
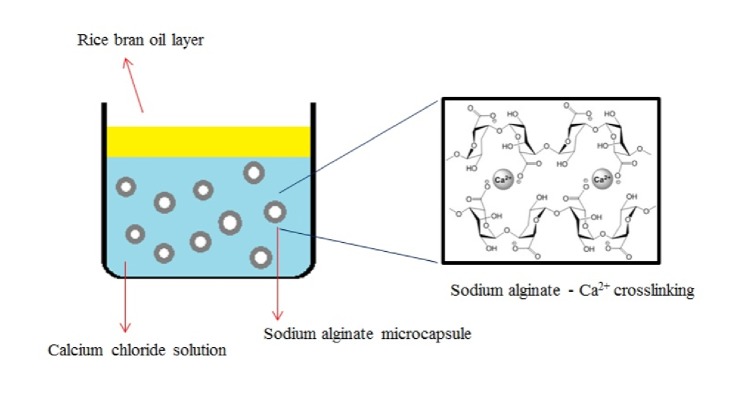
Sodium alginate crosslinking.

When Span 80 and Lecithin were used the results were the same compared to absence of surfactant and no formation of microcapsules (data not shown). The emulsion was more stable than without surfactant but despite increasing the concentrations of both types of surfactants we were not able to prepare the particles. Span 80 and Lecithin are low hydrophilic lipophilic balance (HLB) type surfactants that can produce stable water-in-oil (w/o) emulsion but after mixing with calcium chloride solution microcapsules cannot form as phase inversion cannot be induced. On the other hand, Tween 80 has a high HLB value and readily forms an oil-in-water (o/w) emulsion and inverted from a w/o emulsion when calcium chloride solution was mixed [8–10]. Phase inversion is an important mechanism to change the internal phase in the calcium chloride solution. In our study, when phase inversion occurred, sodium alginate was pushed out from the w/o emulsion to the calcium chloride solution. Calcium ions diffuse through the sodium alginate polymer and the cross-linking between the carboxylic groups of the polymer chains with calcium ions hardens the polymer shell and solidifies it to a microcapsule (Figure 2) [11–14].

The effects of different surfactant concentrations on microcapsule formation were studied. At 1% Tween 80 (Figure 1(A1)) microcapsules had a small size but the particles occurred rarely. This might be due to the fact that there are too few surfactants to surround the internal phase and thus particle preparation was similar to the instance when no surfactant was used. Using 2% and 3 % Tween 80 surfactant concentrations ((Figures 1(A2)-1(A3)) drop-shaped particles could be prepared without any size difference between the two concentrations. As the concentration of the surfactant increased surface tension was reduced and a stable emulsion could be produced. When the concentration of surfactant increased further to 5% (Figure 1(A4)) the particles became smaller with a more spherical shape compared to 2% and 3% Tween 80 at similar particle numbers. At higher surfactant concentrations, the surfactant will evenly surround the internal phase and reduce surface tension to maintain thermodynamic balance. As the aim in the present study was to encapsulate living cells and a high surfactant concentration is known to be toxic to encapsulated cells, the lowest effective concentration for particle preparation was chosen at 2% Tween 80 [6, 15].

Oil and aqueous phase ratios, at 1:1 and 1:2, showed similar results and were both unable to form microcapsules gel layer formation around the emulsion was seen instead around microcapsules (Table 1). When we reduced the aqueous phase to 2:1 and 4:1, these ratios could prepare microcapsules. However, the ratio 2:1 resulted in a higher number of particles compared to the 4:1 ratio as there was more oil phase has a higher viscosity that cause hard to produce the particles and the particles are less precipitated. Microcapsule size and morphology were not different with particles drop shaped at both ratios and an average size of about 300 micrometers. The percentage of entrapped living cells determined by the PrestoBlue assay was calculated at 52.4%.

**Figure 3:**
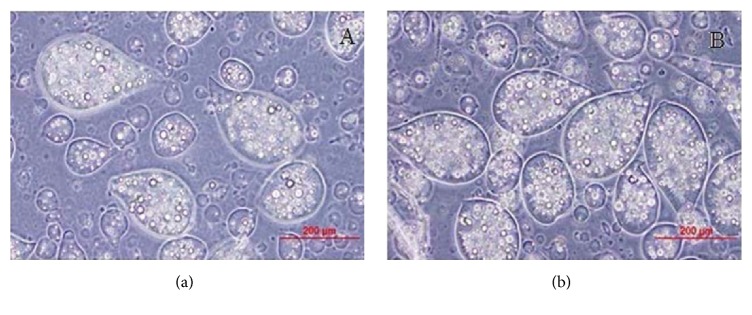
Microcapsule morphology under light microscope incubated in cell culture medium at 37°C on day 0 (a) and day 30 (b).

Then we randomized the microcapsules and observed them at various dates of incubation confirming microcapsule stability for at least 30 days with unaltered morphology and statistically non-significant size difference (Figure 3).

**Figure 4:**
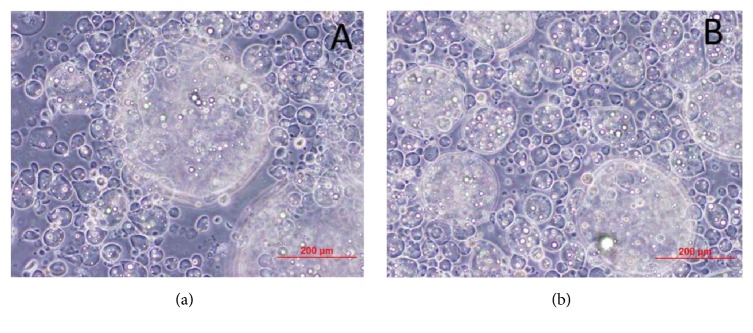
Encapsulated cells under microscope (a) before and (b) after no. 18 needle push pass test. No microcapsule breakage was observed after the test.

Furthermore, we evaluated the stability of the microcapsules in a push pass test through a no. 18 needle and found no breakage of the microcapsules at microscopy observation (Figure 4). We noticed though that as the particles had a high size distribution (Poly Disperse Index: PDI), some were too large to pass through. This observation warrants further studies to reduce the particle size distribution for a more convenient clinical use.

### 3.2. Cell Viability

**Figure 5:**
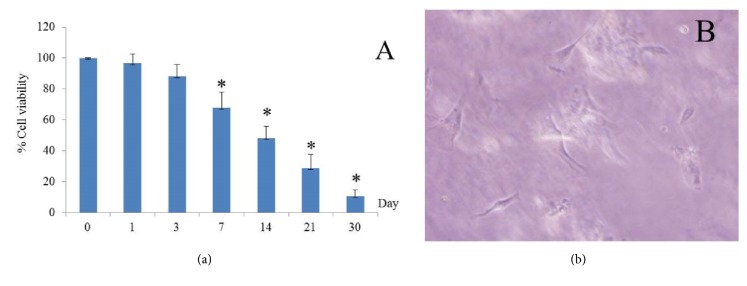
Cell viability.

Emulsion cross-linking encapsulated cells showed continuously decreasing viability that became statistically significant on day 7 (Figure 5(a)). On day 7 some broken microcapsules were encountered and a very small number of cells growing on the well plate could be seen (Figure 5(b)) that disappeared on the following day possibly due to cell damage caused by the microcapsule.

Cell viability continued to decline until day 30 when less than 20% of the encapsulated cells were still viable. A possible reason leading to cell death is a diffusion barrier created by the microcapsule, this being an important factor as cells in the microcapsule need to exchange nutrition, growth factor, and waste and impediment of these processes might be a reason for increased cell death [6, 7, 16–18]. Another potential reason for cell death could be the microcapsule being too densely populated thus waste accumulation along with a diffusion barrier could lead to an increased apoptosis rate [6, 19]. When comparing emulsion cross-linking microcapsule preparation to ionic gelation, the smaller cell proliferation area and longer preparation time of the former could lead to cell weakness and injury. Nevertheless, cell survival was still at 50% on day 14 but rapidly declined thereafter. Reducing particle size and improving particle quality, nutrient exchange and waste product control may increase cell viability and facilitate the use of this method in the clinic.

## 4. Conclusion

In conclusion, emulsion cross-linking was used to prepare microcapsules for human fibroblast encapsulation. Tween 80 surfactant was employed at a 2% concentration resulting from the phase inversion phenomenon in the most efficient and stable size, morphology, and particle numbers without being toxic to the encapsulated cells that showed at least 50% survival on day 14. Emulsion cross-linking microcapsule preparation results in smaller and possibly more diverse particles that can be developed clinically to deliver encapsulated mammalian cells for future disease treatments.

## Figures and Tables

**Table 1:** Effect of oil and aqueous phase ratio on microcapsule formation.

Ratio (oil: aqueous)	Size (*µ*m) ± SD	Poly disperse index (PDI)
1:2	No microcapsule formed	No microcapsule formed
1:1	No microcapsule formed	No microcapsule formed
2:1	328 ±14.7	4.12
4:1	294 ±15.3	3.94

## Data Availability

All data presented have been manually entered in datasets and are available from our first and corresponding authors for inspection upon request.
